# Association of treatment delay and stage with mortality in breast cancer: a nationwide cohort study in Taiwan

**DOI:** 10.1038/s41598-022-23683-y

**Published:** 2022-11-07

**Authors:** Nai-Chen Shih, Pei-Tseng Kung, Wei-Yin Kuo, Wen-Chen Tsai

**Affiliations:** 1 Department of Family Medicine, Taichung Veterans General Hospital, Taichung, Taiwan, R.O.C.; 2 Department of Public Health, China Medical University, Taichung, Taiwan, R.O.C.; 3 Institute of Medicine, Chung Shan Medical University, Taichung, Taiwan, R.O.C.; 4 Department of Healthcare Administration, Asia University, Taichung, Taiwan, R.O.C.; 5 Department of Medical Research, China Medical University Hospital, China Medical University, Taichung, Taiwan, R.O.C.; 6 Department of Health Services Administration, China Medical University, Taichung, Taiwan, R.O.C. No. 100, Sec. 1, Jingmao Rd., Beitun Dist., 406040

**Keywords:** Cancer, Health care, Medical research, Oncology, Risk factors

## Abstract

Breast cancer is the fifth leading cause of cancer death globally. In this retrospective study, we investigated the effects of the diagnosis-to-first-treatment interval (DFTI) and other related factors on cancer-specific survival in patients with breast cancer. We included 49,426 patients newly diagnosed as having breast cancer during 2011–2017. The Cox proportional hazards model was used to analyze the hazard ratio (HR) for mortality with various DFTIs; the HRs of the 31–60-, 61–90-, and ≥ 91-day DFTI groups did not differ significantly compared with the reference group (DFTI ≤ 30 days). After stratifying the patients according to initial tumor stage and age, we found that patients aged 55–64 and ≥ 65 years with stage II breast cancer treated ≥ 91 days after diagnosis had a 3.34- and 2.93-fold higher mortality risk (95% confidence intervals [CIs] 1.29–8.69 and 1.06–8.10, respectively). Patients aged ≥ 65 years with stage IV breast cancer treated within 61–90 or ≥ 91 days after diagnosis had a 7.14- and 34.78-fold higher mortality risk (95% CIs 1.28–39.82 and 3.08–393.32, respectively). In conclusion, DFTI is associated with mortality in patients with stage II and IV breast cancer, especially at an older age.

## Introduction

In 2020, breast cancer was the fifth leading cause of cancer death globally; it was responsible for 684,996 deaths, accounting for approximately 6.9% of cancer deaths worldwide^1^. In 2019, nearly 14,856 women were diagnosed as having breast cancer, and 2633 women died of breast cancer in Taiwan, making it the second leading cause of cancer death among Taiwanese women^2^.

According to the National Cancer Institute’s Surveillance, Epidemiology, and End Results Program data, women diagnosed as having breast cancer between 2011 and 2017 had a 5-year relative survival at localized breast cancer diagnosis was nearly 99.0%^3^. In women with breast cancer exhibiting regional lymph node metastasis and distant metastasis, the 5-year relative survival rates were approximately 85.8% and 29.0%, respectively^3^. Taiwan’s Ministry of Health and Welfare provides free mammography to women who meet certain criteria to ensure the early detection and treatment of breast cancer. However, a recent study indicated that delays and refusals of breast cancer treatment remain prevalent in Taiwan^4^.

Treatment delay influences the overall survival of patients with breast cancer considerably^5^. After a breast cancer diagnosis, patients are typically concerned about treatment time and its influence on their survival^6^. Some studies have revealed that a short DFTI could positively influence disease progression, survival rates, and quality of life^7,8^. However, other studies have indicated that DFTI does not influence patient survival^9–11^. One study revealed that delays in receiving treatment for metastatic breast cancer may be related to adverse survival outcomes^5^; however, other studies have indicated that the mortality risk increases when treatment is delayed in the early stages^6,12^. In the current study, we investigated how the DFTI influences survival in patients at different breast cancer stages. The results may aid in determining treatment time and mitigating the risk of poor prognosis due to delayed treatment at different cancer stages and in monitoring other factors associated with survival.

## Results

### Characteristics of patients with breast cancer for different treatment intervals

In this study, we included 49,426 patients who had been diagnosed with breast cancer between 2011 and 2017. Various factors were associated with the interval between diagnosis and treatment initiation. As listed in Table 1, most of the patients had undergone treatment within 30 days of diagnosis (n = 40,010; 80.95%). Patients who earned a lower monthly salary (NTD20,009–NTD22,800), had stage IV breast cancer or a tumor size of 2–5 cm, had received treatment in a district hospital, and had received treatment in a public hospital had a relatively short DFTI (< 30 days; *P*_*s*_ < 0.001). However, with regard to patient age, CCI score, hormone receptor status (ERA, PRA), HER2 status, and number of involved regional lymph nodes, the differences in DFTIs were found to be nonsignificant (*P* > 0.05).

**Table 1:** Descriptive statistics of patients with breast cancer and different DFTIs.

Variables	Total		Interval from cancer diagnosis to treatment								P-value
			≤ 30 days		31–60 days		61–90 days		≥ 91 days		
	N	%	N	%	N	%	N	%	N	%	
Total	49,426	100.00	40,010	80.95	7996	16.18	961	1.94	459	0.93	
**Age (years)**											0.062
20–54	25,888	52.38	20,924	80.83	4243	16.39	500	1.93	221	0.85	
55–64	13,911	28.15	11,210	80.58	2291	16.47	268	1.93	142	1.02	
≥ 65	9627	19.48	7876	81.81	1462	15.19	193	2.00	96	1.00	
Mean ± SD	54.61 ± 11.72		54.65 ± 11.75		54.31 ± 11.57		54.96 ± 11.83		55.61 ± 11.42		
**Monthly salary (NTD)**											** < 0.001**
< 20,008	12,652	25.60	10,239	80.93	2007	15.86	265	2.09	141	1.11	
20,009–22,800	13,706	27.73	11,245	82.04	2094	15.28	261	1.90	106	0.77	
22,801–38,200	10,698	21.64	8633	80.70	1764	16.49	193	1.80	108	1.01	
> 38,200	12,370	25.03	9893	79.98	2131	17.23	242	1.96	104	0.84	
**CCI score**^a^											0.519
0	28,394	57.45	22,932	80.76	4644	16.36	555	1.95	263	0.93	
1	13,093	26.49	10,681	81.58	2061	15.74	237	1.81	114	0.87	
2	4349	8.80	3493	80.32	722	16.60	89	2.05	45	1.03	
≥ 3	3590	7.26	2904	80.89	569	15.85	80	2.23	37	1.03	
**ERA**											0.099
Negative	10,492	21.23	8564	81.62	1651	15.74	196	1.87	81	0.77	
Positive	38,934	78.77	31,446	80.77	6345	16.30	765	1.96	378	0.97	
**PRA**											0.629
Negative	14,860	30.07	12,065	81.19	2382	16.03	286	1.92	127	0.85	
Positive	34,566	69.93	27,945	80.85	5614	16.24	675	1.95	332	0.96	
**HER2**											0.413
Negative	11,024	22.30	8927	80.98	1793	16.26	195	1.77	109	0.99	
Positive	38,402	77.70	31,083	80.94	6203	16.15	766	1.99	350	0.91	
**Tumor size**											** < 0.001**
< 2 cm	22,590	46.05	18,060	79.95	3767	16.68	515	2.28	248	1.10	
2–5 cm	22,092	45.04	18,142	82.12	3426	15.51	368	1.67	156	0.71	
> 5 cm	4373	8.91	3493	79.88	762	17.43	70	1.60	48	1.10	
Missing	371										
**No. of lymph node involvement**											0.408
0	30,092	63.31	24,274	80.67	4923	16.36	608	2.02	287	0.95	
1–3	10,899	22.93	8855	81.25	1756	16.11	194	1.78	94	0.86	
4–9	3957	8.32	3241	81.91	615	15.54	66	1.67	35	0.88	
≥ 10	2586	5.44	2120	81.98	397	15.35	49	1.89	20	0.77	
Missing	1892										
**Cancer stage**											** < 0.001**
Stage I	19,780	40.02	15,850	80.13	3255	16.46	451	2.28	224	1.13	
Stage II	20,091	40.65	16,380	81.53	3206	15.96	350	1.74	155	0.77	
Stage III	7974	16.13	6450	80.89	1311	16.44	142	1.78	71	0.89	
Stage IV	1581	3.20	1330	84.12	224	14.17	18	1.14	9	0.57	
**Treatment**											** < 0.001**
Surgery	3292	6.66	2,520	76.55	571	17.35	118	3.58	83	2.52	
Surgey + chemo	14,463	29.26	11,226	77.62	2808	19.42	319	2.21	110	0.76	
Surgey + chemo + TT	4087	8.27	3399	83.17	655	16.03	30	0.73	3	0.07	
Surgey + chemot + HT	3757	7.60	3366	89.59	361	9.61	26	0.69	4	0.11	
Surgery + HT	6557	13.27	4987	76.06	1281	19.54	192	2.93	97	1.48	
Surgery + HT + radiotherapy	5955	12.05	4697	78.87	1073	18.02	152	2.55	33	0.55	
Surgery + others	4736	9.58	4201	88.70	478	10.09	36	0.76	21	0.44	
Chemo	2168	4.39	1729	79.75	367	16.93	52	2.40	20	0.92	
Chemo + TT	1065	2.15	720	67.61	304	28.54	29	2.72	12	1.13	
Others	3346	6.77	3165	94.59	98	2.93	7	0.21	76	2.27	
**Hospital level**											** < 0.001**
Medical center	27,360	55.36	21,396	78.20	5017	18.34	664	2.43	283	1.03	
Regional hospital	20,855	42.19	17,540	84.10	2871	13.77	279	1.34	165	0.79	
District hospital	1211	2.45	1074	88.69	108	8.92	18	1.49	11	0.91	
**Hospital ownership**											** < 0.001**
Public	33,813	68.41	27,892	82.49	5125	15.16	520	1.54	276	0.82	
Non-public	15,613	31.59	12,118	77.61	2871	18.39	441	2.82	183	1.17	

*NTD* New Taiwan Dollar (USD1 ≈ NTD30), *CCI* Charlson comorbidity index, *ERA* estrogen receptor assay, *PRA* progesterone receptor assay, *HER2* human epidermal growth factor receptor 2, *RT* radiotherapy, *Chemo* chemotherapy, *HT* hormone therapy, *TT* targeted therapy.

^a^Exclusion of cancer from the CCI score.

Significant P values (< 0.05) are highlighted in bold.

### Influence of DFTI and other related factors on mortality risk

A Cox proportional hazards model was used to examine the relationship of patient survival with the relevant variables (Table 2). The DFTI, hospital level, and hospital ownership were nonsignificant predictors of mortality. ER, PR, and HER2 status were protective factors for mortality risk (adjusted HRs [95% CIs] 0.64 [0.56–0.72], 0.51 [0.45–0.57], and 0.88 [0.80–0.97], respectively; all P_s_ < 0.05) after other related factors were controlled for. Tumor size, number of involved lymph nodes, and cancer stage also were positively correlated with mortality risk (P < 0.05).

**Table 2:** Influence of DFTIs and other related factors on mortality risk in patients with confirmed breast cancer.

Variables	Total		Alive		Death		P-value	Adjusted model^2^		
	N	%	N	%	N	%		HR	95% CI	P-value
Total	49,426	100.00	46,835	94.76	2591	5.24				
**Duration**							< 0.001			
30 days	40,010	80.95	37,844	94.59	2166	5.41		1.00		
31–60 days	7996	16.18	7646	95.62	350	4.38		1.01	0.90–1.14	0.895
61–90 days	961	1.94	917	95.42	44	4.58		1.03	0.74–1.43	0.875
≥ 91 days	459	0.93	428	93.25	31	6.75		1.41	0.96–2.08	0.077
**Age (years)**							< 0.001			
20–54 (Ref.)	25,888	52.38	24,724	95.50	1164	4.50		1.00		
55–64	13,911	28.15	13,215	95.00	696	5.00		0.88	0.79–0.98	**0.024**
≥ 65	9627	19.48	8896	92.41	731	7.59		1.28	1.12–1.46	** < 0.001**
Mean ± SD	54.61 ± 11.72		54.44 ± 11.59		57.70 ± 13.39					
**Monthly salary (NTD)**							< 0.001			
< 20,008 (Ref.)	12,652	25.60	11,740	92.79	912	7.21		1.00		
20,009–22,800	13,706	27.73	13,008	94.91	698	5.09		0.86	0.77–0.96	**0.006**
22,801–38,200	10,698	21.64	10,200	95.34	498	4.66		0.86	0.76–0.97	**0.011**
> 38,200	12,370	25.03	11,887	96.10	483	3.90		0.86	0.76–0.97	**0.013**
**CCI score**^**1**^							< 0.001			
0 (Ref.)	28,394	57.45	26,970	94.98	1424	5.02		1.00		
1	13,093	26.49	12,441	95.02	652	4.98		1.04	0.94–1.15	0.463
2	4349	8.80	4108	94.46	241	5.54		1.10	0.94–1.27	0.235
≥ 3	3590	7.26	3316	92.37	274	7.63		1.31	1.13–1.52	** < 0.001**
**ERA**							< 0.001			
Negative (Ref.)	10,492	21.23	9483	90.38	1009	9.62		1.00		
Positive	38,934	78.77	37,352	95.94	1582	4.06		0.64	0.56–0.72	** < 0.001**
**PRA**							< 0.001			
Negative (Ref.)	14,860	30.07	13,513	90.94	1347	9.06		1.00		
Positive	34,566	69.93	33,322	96.40	1244	3.60		0.51	0.45–0.57	** < 0.001**
**HER2**										
Negative (Ref.)	11,024	22.30	10,418	94.50	606	5.50		1.00		
Positive	38,402	77.70	36,417	94.83	1985	5.17		0.88	0.80–0.97	**0.012**
**Tumor size**							< 0.001			
< 2 cm (Ref.)	22,590	46.05	22,272	98.59	318	1.41		1.00		
2-5 cm	22,092	45.04	20,783	94.07	1309	5.93		1.75	1.49–2.07	** < 0.001**
> 5 cm	4373	8.91	3494	79.90	879	20.10		2.90	2.43–3.47	** < 0.001**
**No. of lymph node involvement**							< 0.001			
0 (Ref.)	30,092	63.31	29,448	97.86	644	2.14		1.00		
1–3	10,899	22.93	10,287	94.38	612	5.62		1.77	1.56–2.00	** < 0.001**
4–9	3957	8.32	3470	87.69	487	12.31		1.87	1.60–2.19	** < 0.001**
≥ 10	2586	5.44	1964	75.95	622	24.05		3.22	2.77–3.75	** < 0.001**
**Cancer stage**							< 0.001			
Stage I (Ref.)	19,780	40.02	19,594	99.06	186	0.94		1.00		
Stage II	20,091	40.65	19,418	96.65	673	3.35		1.81	1.44–2.26	** < 0.001**
Stage III	7974	16.13	6880	86.28	1094	13.72		4.11	3.19–5.28	** < 0.001**
Stage IV	1581	3.20	943	59.65	638	40.35		13.60	10.46–17.68	** < 0.001**
**Treatment**							< 0.001			
Surgery (Ref.)	3292	6.66	3064	93.07	228	6.93		1.00		
Surgery + chemo	14,463	29.26	13,583	93.92	880	6.08		0.69	0.59–0.81	** < 0.001**
Surgery + chemo + TT	4087	8.27	3834	93.81	253	6.19		0.43	0.36–0.53	** < 0.001**
Surgery + chemot + HT	3757	7.60	3570	95.02	187	4.98		0.83	0.67–1.03	0.092
Surgery + HT	6557	13.27	6349	96.83	208	3.17		1.04	0.84–1.28	0.731
Surgery + HT + Radiotherapy	5955	12.05	5887	98.86	68	1.14		0.60	0.44–0.81	**0.001**
Surgery + others	4736	9.58	4441	93.77	295	6.23		0.74	0.61–0.90	**0.002**
Chemo	2168	4.39	1950	89.94	218	10.06		0.98	0.80–1.21	0.878
Chemo + TT	1065	2.15	975	91.55	90	8.45		0.60	0.45–0.79	** < 0.001**
Others	3346	6.77	3182	95.10	164	4.90		0.90	0.71–1.13	0.367
**Hospital level**							0.1359			
Medical center (Ref.)	27,360	55.36	25,961	55.43	1399	53.99		1.00		
Regional hospital	20,855	42.19	19,735	42.14	1120	43.23		1.01	0.93–1.11	0.778
District hospital	1211	2.45	1,139	2.43	72	2.78		1.00	0.77–1.28	0.969
**Hospital ownership**							0.7056			
Public (Ref.)	33,813	68.41	32,043	68.42	1770	68.31		1.00		
Non-public	15,613	31.59	14,792	31.58	821	31.69		0.95	0.86–1.04	0.235

*NTD* New Taiwan Dollar (USD1≈ NTD30), *CCI* Charlson comorbidity index, *ERA* estrogen receptor assay, *PRA* progesterone receptor assay, *HER2* human epidermal growth factor receptor 2, *RT* radiotherapy, *Chemo* chemotherapy, *HT* hormone therapy, *TT* targeted therapy.

^1^Exclusion of cancer from the CCI score.

^2^The Cox proportional hazard model was also controlled for relevant variables including marital status, education level, and level of urbanization.

Significant P values (< 0.05) are highlighted in bold.

### Mortality risk in patients with delayed treatment according to the different ages and various breast cancer stages

**Figure 1:**
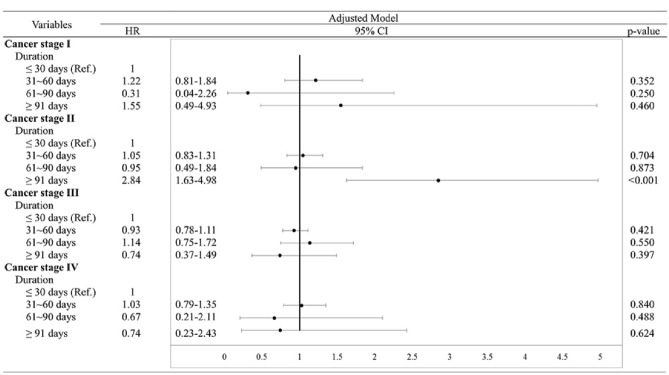
Influence of DFTI on mortality risk in patients at various breast cancer stages. The related variables (age, marital status, education level, monthly salary, level of urbanization, CCI score, tumor size, number of involved lymph nodes, hormone receptor status, HER2 status, treatment type, hospital level, and hospital ownership) were controlled in each model.

**Figure 2:**
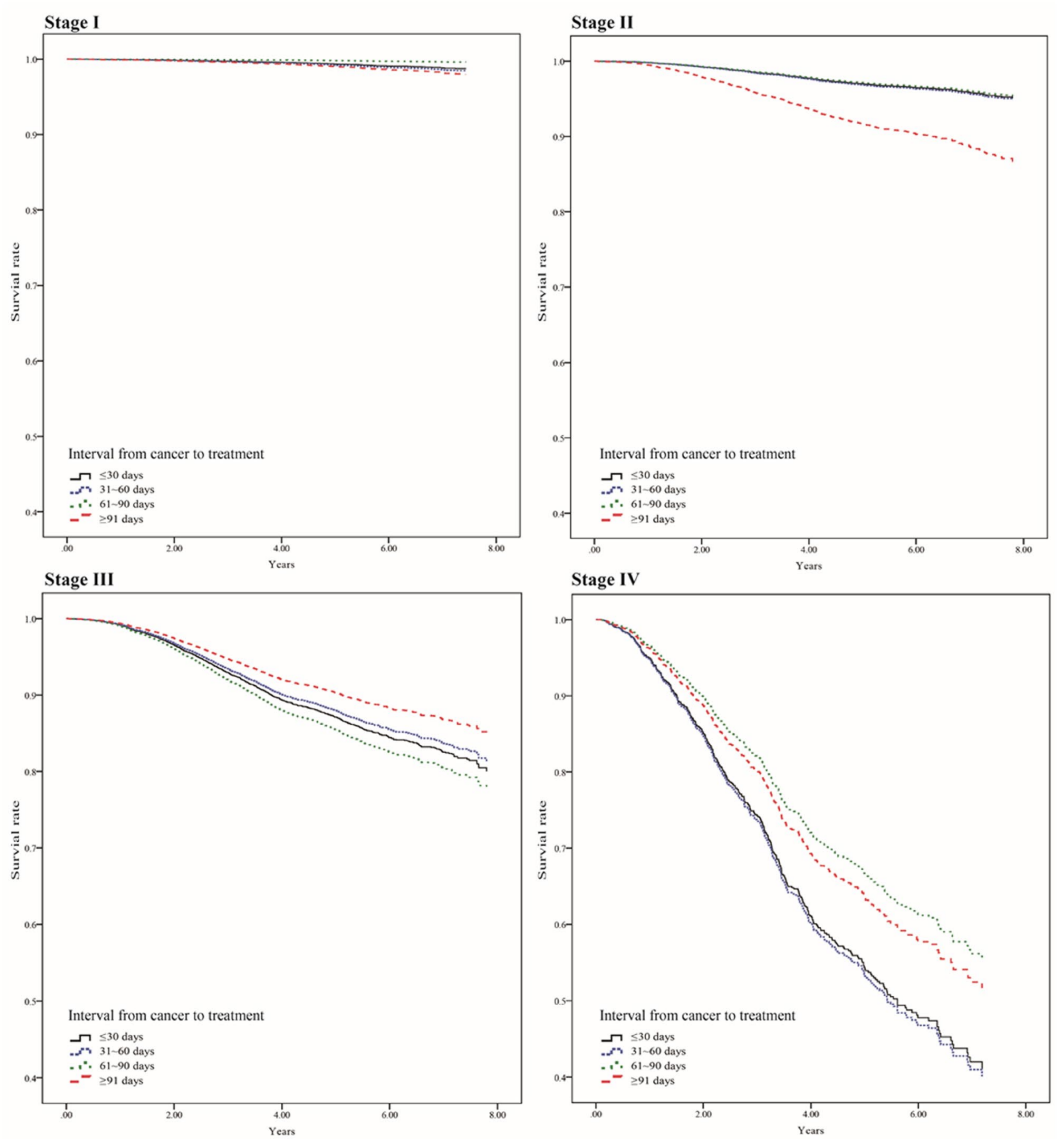
Adjusted survival curves of patients with different breast cancer stages and DFTIs. The related variables (age, marital status, education level, monthly salary, level of urbanization, CCI score, tumor size, number of involved lymph nodes, hormone receptor status, HER2 status, treatment type, hospital level, and hospital ownership) were controlled.

Figure 1 lists the mortality risk after stratifying the patients according to initial tumor stage with different DFTIs. In patients at stages I, III, and IV, a DFTI of > 30 days did not have a significant effect on mortality compared with the reference group (DFTI ≤ 30 days). However, patients at stage II with a ≥ 91-day DFTI showed a 2.84-fold increase in mortality risk compared with the reference group (DFTI ≤ 30 days; 95% CI 1.63–4.98, *P* < 0.001). As illustrated in the adjusted survival curves (Fig. 2), after stratification according to initial tumor stage, DFTIs exhibited no significant difference compared with the reference group (DFTI ≤ 30 days) in all patients except for those at stage II. The DFTI remained a significant prognosticator in patients at stage II who started treatment ≥ 91 days after diagnosis (Fig. 2). Moreover, we analyzed the impact of DFTIs on mortality risk according to age and cancer stage with multivariate Cox proportional hazard models (Table 3). We determined that compared with the reference group (DFTI ≤ 30 days), stage II patients with a ≥ 91-day DFTI were 3.34 and 2.93 times more likely to die at the ages of 55–64 and ≥ 65, respectively (95% CIs 1.29–8.69, *P* = 0.013, and 1.06–8.10, *P* = 0.039, respectively). Moreover, compared with the reference group (DFTI ≤ 30 days), stage IV patients at the age of ≥ 65 with a 61–90 and > 91-day DFTI had a 7.14- and 34.78-fold increased mortality risk (95% CIs  1.28–39.82, *P* = 0.025, and 3.08–393.32, *P* = 0.004, respectively).

**Table 3:** Influence of DFTI on mortality risk in patients of different ages and at various breast cancer stages.

Variables	Patients aged 20–54					Patients aged 55–64					Patients aged ≥ 65				
	HR	95% CI			P-value	HR	95% CI			P-value	HR	95% CI			P-value
**Cancer stage I**^**1**^															
**Duration**															
≤ 30 days (Ref.)	1.00					1.00					1.00				
31–60 days	0.97	0.47	–	2.01	0.941	1.50	0.69	–	3.25	0.307	1.29	0.65	–	2.54	0.470
61–90 days	1.07	0.15	–	7.85	0.950	–		–		–	–		–		–
≥ 91 days	2.03	0.27	–	15.32	0.492	1.68	0.22	–	13.16	0.620	1.72	0.22	–	13.30	0.605
**Cancer stage II**^**1**^															
**Duration**															
≤ 30 days (Ref.)	1.00					1.00					1.00				
31–60 days	0.98	0.70	–	1.39	0.921	0.81	0.50	–	1.34	0.412	1.30	0.89	–	1.90	0.181
61–90 days	0.95	0.35	–	2.56	0.913	1.60	0.50	–	5.13	0.431	0.61	0.15	–	2.48	0.492
≥ 91 days	2.47	0.90	–	6.81	0.080	3.34	1.29	–	8.69	**0.013**	2.93	1.06	–	8.10	**0.039**
**Cancer stage III**^**1**^															
**Duration**															
≤ 30 days (Ref.)	1.00					1.00					1.00				
31–60 days	0.87	0.67	–	1.13	0.308	0.90	0.61	–	1.32	0.578	1.07	0.76	–	1.50	0.712
61–90 days	0.77	0.39	–	1.53	0.460	1.36	0.57	–	3.23	0.489	1.76	0.87	–	3.55	0.114
≥ 91 days	0.67	0.27	–	1.65	0.385	0.66	0.09	–	4.92	0.687	1.06	0.26	–	4.33	0.935
**Cancer stage IV**^**1**^															
**Duration**															
≤ 30 days (Ref.)	1.00					1.00					1.00				
31–60 days	0.95	0.63	–	1.43	0.809	1.18	0.73	-	1.92	0.501	0.77	0.39	–	1.55	0.467
61–90 days	0.28	0.04	–	2.03	0.206	–		–		–	7.14	1.28	–	39.82	**0.025**
≥ 91 days	0.54	0.07	–	4.17	0.552	0.43	0.05	–	3.81	0.447	34.78	3.08	–	393.32	**0.004**

^1^The related variables (marital status, education level, monthly salary, level of urbanization, CCI score, tumor size, number of involved lymph nodes, hormone receptor status, HER2 status, treatment type, hospital level, and hospital ownership) were controlled in each model.

Significant P values (< 0.05) are highlighted in bold.

## Discussion

In this study, we analyzed 49,426 patients with new-onset breast cancer and found no statistically significant differences between early treatment and survival. However, stratified analysis by age and stage demonstrated that stage II patients aged > 55 years with a ≥ 91-day DFTI and stage IV patients aged ≥ 65 years with a > 60-day DFTI had an elevated mortality risk.

Some studies have explored the impact of delayed treatment on mortality risk in patients with breast cancer, but they have not reported any conclusive results. Yoo et al. demonstrated that a delay in treatment initiation with a cutoff value of 15, 30, 45, and 60 days after biopsy confirmation did not affect disease-free and overall breast cancer survival^11^. Mujar et al., who also reported that a DFTI of > 2 weeks, 1 month, and 2 months did not affect survival^10^. However, several studies have demonstrated the opposite. In female patients with breast cancer whose first treatment was surgery, the 5-year survival rate was significantly higher in patients with a < 2-week DFTI compared with those with a > 6-week DFTI (90% vs. 80%; *P* < 0.05)^18^. Yun et al. demonstrated that the 5-year survival rate in patients with breast cancer and a DFTI of > 1 month was low (HR 1.59; 95% CI 1.37–1.84)^19^. According to the National Breast and Cervical Cancer Early Detection Program, the DFTI should not exceed 60 days^20^.

Studies have reported the impact of delayed treatment on patient survival in various subgroups. McLaughlin et al. found that a ≥ 60-day DFTI substantially increased the mortality risk of patients at a late cancer stage but did not affect the overall survival of patients at an early stage^21^. Our results revealed that at stage IV, a higher risk existed between treatment delay and breast cancer-related mortality among people aged ≥ 65 years. Nonetheless, the results accord with those of previous studies showed that the overall mortality HR significantly increased for each increasing interval at cancer stages I and II^6^. Besides, Khorana et al. reported that an increased DFTI is associated with worsened survival in patients with stage I and II breast cancer but that it is associated to a much lower degree with outcomes in patients with stage III breast cancer^12^. This result suggests that cancer stage is an important prognostic factor for survival response after delayed treatment. Thus, we further evaluated the association between treatment delay and cancer stage at different ages and breast cancer mortality. A study reported that compared with older women with breast cancer, younger women with breast cancer are more likely to have higher-grade, larger tumors with ER/PR negativity and lymph node positivity; moreover, their mortality risk is higher^22^. That study also revealed that compared with older women, younger women are more likely to die if diagnosed as having stage I or II disease and less likely to die if diagnosed as having stage IV disease^22^. However, the database used in the aforementioned study did not contain information on comorbidities or other cancer treatments, such as chemotherapy and endocrine therapy. Patients who are younger and have late-stage disease may be undergoing more aggressive treatment than older women at the same stage due to the lack of comorbidities. In the current study, we collected complete data on patient treatments and comorbidities. Women aged 55–64 years exhibited significantly less mortality risk than those aged 20–54 years (HR 0.88; 95% CI 0.79–0.98).

Previous research showed that the lower the income of a patient was, the later the stage at which a patient’s cancer was diagnosed^23^. A study also revealed that patients at a later cancer stage with a lower socioeconomic status experience an especially longer DFTI and poorer prognosis than do those with a higher socioeconomic status^24^. Although the quality of medical services has been improved in Taiwan after the introduction of National Health Insurance in recent years, patients with higher incomes still have access to more treatment options than do those with lower incomes. A study indicated that the presence of comorbid diseases at breast cancer diagnosis was an independent adverse prognostic factor for mortality in patients with breast cancer^25^, which accords with the current results. In breast cancer, immunohistochemistry subtypes, together with grade, tumor size, and nodal status, are related to survival. These factors were previously found to be independent predictors of breast cancer mortality: the mortality risk was 20–40-fold higher in patients with the worst prognosis than in those with tumors having smaller size, lower grade, and ER(+)/PR(+)/HER2(−) status^26^. Women who initially visited a teaching hospital also had significantly better survival than did those who initially visited a community hospital^27^.

The strengths of our study, compared with previous studies, are related to the sample size, data source, and breast cancer stage analysis. We used nationwide data from the Taiwan Cancer Registry to prevent any selection bias, which may exist in traditional hospital-based observational studies. Our study also examined the impact of the DFTI on the survival rates of patients at different breast cancer stages and different age subgroups. The databases we included contain complete information on the factors that may also influence breast cancer mortality.

This study, however, has several limitations. We evaluated the effects of immunohistochemistry subtype, tumor size, and nodal spread on breast cancer mortality; however, our collected data did not include Ki67 status, and therefore, we could not analyze the combined effects of the aforementioned factors (i.e., hormone receptor status, HER2 status and Ki67 status) on breast cancer death. Moreover, our results cannot be generalized to patients in other countries, mainly because the National Health Insurance system has improved health care access and cost in Taiwan, enabling Taiwan’s residents to receive breast cancer treatments without a copayment. Finally, in the current study, 8167 patients of 81,906 included patients—roughly 9% of the total study population—did not have cancer stage information, and therefore, they were excluded from our study participants. Nonetheless, because this study included a large nationwide population, we believe that a negligible number of women with an unknown cancer stage were excluded.

## Conclusions

The results of this study highlight how the cancer stage and age at diagnosis affect survival among patients with different DFTIs. To increase the timeliness of receiving treatment, we suggest that interventions should especially be targeted at older patients with stage II and IV breast cancer.

## Materials and methods

### Study design

This retrospective cohort study was conducted to evaluate the influence of the DFTI and other related factors on mortality among female patients with breast cancer. Confirmed breast cancer cases diagnosed between 2011 and 2017 were sampled, and their survival was followed until the end of 2018. The start date of follow-up was the date of first diagnosis, and the end date was the date of loss to follow-up, death, or the end of the study period (December 31, 2018), whichever occurred first.

### Data sources

To select our sample participants, we accessed the Taiwan Cancer Registry, published by the Health Promotion Administration, Ministry of Health and Welfare. The Taiwan Cancer Registry is a nationwide population-based cancer registry system that was established in 1979. The database includes detailed information on cancer staging, cancer site-specific factors, treatment, and recurrence^13^. In addition, we connected these data to 2009–2018 data from the National Health Insurance Research Database (NHIRD), the Registry for Catastrophic Illness Patients Database (RCIPD), and the Cause of Death File, Ministry of Health and Welfare. The RCIPD is a subdatabase of the NHIRD, which contains the data of 99.99% of Taiwan’s population^14^. The RCIPD includes the health-care data of patients diagnosed as having any of the 30 specified catastrophic illnesses (such as malignancies, severe hereditary diseases, immune diseases or disorders)^15^. People with any catastrophic illness are exempt from consultation, pharmacy, treatment, and hospitalization fees under National Health Insurance. We extended 2 years of data (2009 and 2010) from the NHIRD to evaluate the Charlson comorbidity index (CCI) scores and other catastrophic illnesses of the included patients. This study was approved by the institutional review board of Cheng Ching Hospital (IRB number: HP150004) and was conducted in accordance with the Helsinki Declaration. The informed consent was waived by the Research Ethnics Committee of China Medical University Hospital.

### Study participants

**Figure 3:**
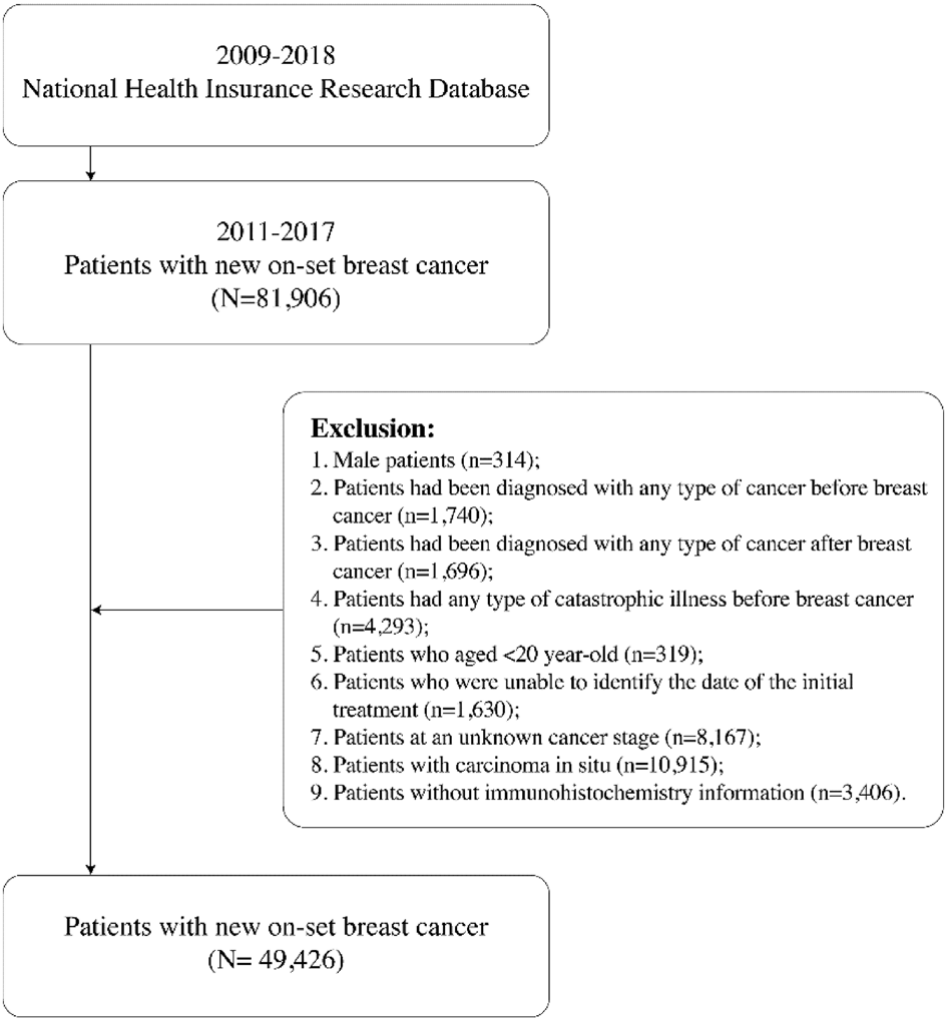
Study participant selection process.

In total, 81,906 patients were newly diagnosed as having breast cancer between 2011 and 2017. Their diagnosis was defined on the basis of International Classification of Diseases for Oncology, Third Edition codes C50.0–C50.9. Of all patients, only 49,426 patients were included for further analysis (Fig. 3) after the following patients were excluded: male patients, patients diagnosed as having any type of cancer before or after breast cancer diagnosis, patients having any type of catastrophic illness before breast cancer, patients aged < 20 years, patients without a known date of initial treatment, patients at an unknown cancer stage, patients with carcinoma in situ, and patients without any immunohistochemistry information.

### Variable definitions and descriptions

We defined DFTI as the interval between the date of breast cancer diagnosis based on biopsy and the date on which the first treatment was initiated. Four DFTI groups were used to divide the included patients: ≤ 30, 31–60, 61–90, and ≥ 91 days. Next, we defined age as the age at which a patient received a confirmed breast cancer diagnosis based on pathological findings. We categorized marital status as single, married, divorced, widowed, and missing (people with unknown marital status) and grouped the education levels into six categories. The income of the patients was based on their monthly salary. The environmental factors were based on the urbanization level of the patients’ areas of residence before cancer diagnosis; a total of seven levels—from highly developed urban cities (level 1) to remote districts (level 7)—were employed^16^.

The degree of comorbidity—a weighted index based on the presence of comorbid conditions within 2 years before cancer diagnosis—was classified into four levels based on CCI scores (Deyo’s CCI)^17^. Tumor subtypes were classified on the basis of estrogen receptor, progesterone receptor, and human growth factor/neu receptor status, as recorded according to the pathologists’ interpretation of the assays. The largest dimension of the tumor (in centimeters) as determined through pathology examination were considered the tumor size. Regional lymph nodes were defined as the most proximal lymph nodes serving as immediate drainage sites for the tumors, and these included axillary nodes, ipsilateral intramammary nodes, internal mammary nodes, and supraclavicular nodes. Cancer stages were categorized according to the *American Joint Committee on Cancer Staging Manual*, *Eighth Edition*. The patients were also classified based on the treatment they received within 6 months after breast cancer diagnosis. Other variables included hospital level and treatment hospital ownership.

### Main outcome measurements

The primary outcome was cancer-specific mortality in the patients with breast cancer. Follow-up duration was defined as the duration from the date of diagnosis to the date of death or follow-up endpoint (December 31, 2018). Confirmation of death was made on the basis of the administrative data (Cause of Death File).

### Statistical analysis

For descriptive statistics, we included the basic characteristics, income, environmental factors, health status, tumor characteristics, stage, treatment type, primary hospital information, and DFTI distribution.

The log-rank test and Cox proportional hazards model were adopted for inferential statistics. First, a bivariate analysis was performed using the log-rank test to determine significant differences between the survival status by the end of 2018 and the DFTI or other variables. The adjusted Cox proportional hazards model was used to analyze the relative mortality risk in breast cancer patients with different DFTIs, after the related variables were controlled for. Subsequently, we analyzed the influence of the DFTI on the survival of patients at various cancer stages and of different ages. Finally, we estimated survival time according to the adjusted survival curves for all patients with breast cancer; the stratification analyses by tumor stage were performed to investigate the influences of different DFTIs on patient survival.

In this study, SAS (version 9.4; SAS Institute, Cary, NC, USA) was used for data analysis, with the significance level (α) set at 0.05.

## Data Availability

This study used the National Health Insurance Research Database published by the Ministry of Health and Welfare, Taiwan. Due to legal restrictions imposed by the Taiwan government under the Personal Information Protection Act, the database cannot be made publicly available. All researchers can apply to use the database to conduct their studies. Requests for data can be sent as a formal proposal to the Science Center of the Ministry of Health and Welfare (https://www.mohw.gov.tw/np-108-2.html). Any raw data are not allowed to be brought out from the Science Center. Only the analytic outputs in table or figure format can be printed out. The restrictions prohibit authors from making the minimal data set publicly available.
